# Gender Difference in Falls among Adults Treated in Emergency Departments and Outpatient Clinics

**DOI:** 10.4172/2167-7182.1000152

**Published:** 2014-03-20

**Authors:** Feifei Wei, Amy L Hester

**Affiliations:** 1 Department of Biostatistics, Fay W Boozman College of Public Health, University of Arkansas for Medical Sciences, USA; 2 Director of Clinical Informatics and Innovation, University of Arkansas for Medical Sciences Medical Center, USA

**Keywords:** Fall, Adults, Age, Gender, Injury, Surveillance

## Abstract

**Background:**

This study examined the impact of gender on age-related increase for falls and injurious falls resulting in head injuries/fractures among adults, using data from both emergency department and clinic visits. We also estimated the percentages of falls treated in points of entry outside of emergency departments.

**Methods:**

The study population consisted of 259,611 adults seen at emergency department, inpatient, and/or outpatient facilities between January, 2007 and June, 2012 at a US medical center. Rates of falls and injurious falls with head injuries/fractures were calculated by age and gender.

**Results:**

After using both emergency department and clinic visit data, medically consulted falls and injurious falls resulting in head injuries/fractures increased with age for females aged ≥ 18 years. For males, these rates declined, reached the lowest point at age of 65-74, and then increased again. Thirty-nine percent of females and 63% of males treated their falls in clinics, instead of emergency departments.

**Conclusion:**

Gender disparity of medically consulted falls and related injuries exits among adults. Age and gender targeted fall injury prevention interventions need further development. Significant numbers of fall-related injuries were treated at clinics; future research is needed to determine whether fall injury surveillance should be expanded to include outpatient clinics.

## Introduction

Unintentional falls were the leading cause of nonfatal injuries treated in hospital emergency departments in 2010 [1]. After adjusted for inflation, the 2010 direct medical costs including hospitalizations of falls were $30.0 billion [2,3]. The National Electronic Injury Surveillance System - all injury program (NEISS-AIP) collects data on ED visits from a national probability sample of 66 U.S. hospitals. It estimated that there were total 9.16 million nonfatal fall-related ED visits in 2010, resulting in 861,844 hospitalizations [1]. NEISS-AIP data also has found age and gender differences on medically consulted falls that rates of fall injuries treated in emergency departments rose sharply with age and were higher for women among aged ≥ 65 years [4].

Although age and gender difference have been observed [4,5] little has been reported as to whether the rate of age-related increase for medically consulted falls differs by gender. It is also unknown whether gender differences for medically consulted falls will still be observed, if both fall-related emergency department and outpatient clinic visits have been taken into account. Medically consulted falls represent those falls after which a person received medical care in either an emergency departments or other point of care such as outpatient clinics.

The primary purpose of this article is to examine the impact of gender on age-related increase for both medically consulted falls and injurious falls resulting in head injuries or fractures among patients aged ≥ 18 years, using data from not only emergency department but also outpatient clinic visits. Fractures and head injuries are of particular interest as these injuries more often result in disability and/or institutionalization than other injuries. The results provide critical information for the development of age and gender targeted strategies to reduce fall injuries among US adults. This study also estimates the percentages of medically consulted falls, which were treated in all points of entry including both clinics and emergency departments.

## Methods

The study population consists of all patients aged ≥ 18 years seen at emergency department, inpatient, and/or outpatient facilities at a comprehensive academic medical center in the south central United States between January, 2007 and June, 2012. This medical center is the only adult Level One Trauma Center in that state and has more than 1,100 physicians and over 100 clinics covering every medical specialty.

Data was retrieved from the organization’s data warehouse. This warehouse contains data entered into patient’s medical records from both inpatient and outpatient visits. Outpatient visits include those visits to the emergency department and clinics. The dataset from the warehouse does not distinguish which visits were specifically to the emergency department versus clinics. Furthermore, a clinic visit could be a follow-up from a visit to another emergency department.

To estimate the numbers of ED originated versus clinic originated visits, NEISS-AIP data was utilized. By using the estimates from the NEISS-AIP findings to estimate the number of expected ED visits from the study population, we could then determine the estimated percentage of fall-related visits that had points-of-entry originated in the clinics.

We used the International Classification of Diseases, 9th Revision, Clinical Modification (ICD-9-CM) diagnosis codes of E880-E888.xx to identify medically consulted falls. These E Codes were selected because they do not include those falls that result in injury in occupational settings.

To identify injurious falls with head injuries or fractures, we examined the primary diagnosis of the identified medically consulted falls. If the primary diagnosis of a medically consulted fall was 850-854.xx (intracranial injury), 959.01 (head Injuries), 800-804.xx (fracture of skull), 805-809.xx (fracture of neck/trunk), 810-819.xx (fracture of upper limb), or 820-829.xx (fracture of lower limb), it was classified as an injurious fall with head injury or fracture. These head injuries and fractures were selected because they are the most common and costly of fall related injuries [6]. Rates of medically consulted falls and injurious falls with head injuries or fractures were calculated by age and gender. To calculate the rate of medical consulted falls (or injurious falls), we divided the number of study population patients who had medical consulted falls (or injurious falls) within each age and gender category by the total number of study population patients within that category. Analyses were performed in 2013 using SAS 9.2.

## Results

There were total 259,611 patients in the study population representing all patients treated from January 2007 to June 2012. Two percent of the study population identified themselves as Hispanic; 25% and 68% were black and white, respectively; 47%, 33%, 11%, 6% and 2% were aged 18-44, 45-64, 65-74, 75-84, and 85+ years, respectively; and 60% were female. These age ranges were used as they are the typical cut point for reporting similar data. Table 1 show that among 14,016 patients (5.4%) who had a medical consulted fall, 29% (aged 18-44 years) to 39% (aged 85+) of them suffered fall-related head injuries or fractures.

**Figure 1:**
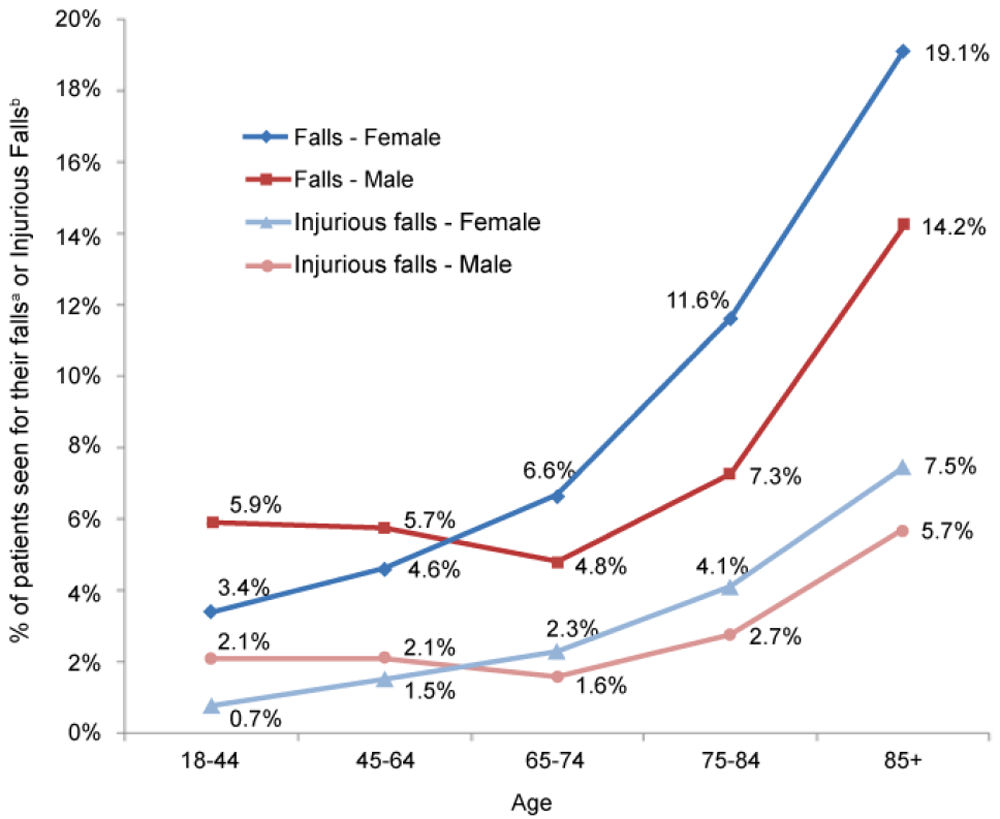
Percentage of patients seen for falls^a^ and injurious falls with head injuries or fractures^b^ in emergency department, inpatient and/or outpatient facilities at an academic medical center in the south central United States, between 01/01/2007 and 06/30/2012, by gender and age.

A total of 6,206 (5.9%) men and 7,810 (5.0%) women in the study population experienced one or more medically consulted falls between January, 2007 and June, 2012. Among them, 2,226 (35.9%) men and 2,358 (30.2%) women had a fall resulting in a head injury or fracture. Figure 1 shows rates of medical consulted falls and injurious falls with head injuries or fractures by gender and age. Rates of medically consulted falls ranged between 3.4% (females aged 18-44 years) and 19.1% (females aged 85+ years). Among these medical consulted falls, 22% (females aged 18-44 years) to 40% (males aged 85+ years) of them were injurious falls that resulted in head injuries or fractures. For females, both medical consulted falls and injurious falls rose with age, and increased sharply on and after age 65. For males, the rates of medically consulted falls and injurious falls reached the lowest point at the age of 65-74, before increasing with age. Males had higher rates of both medical consulted falls and injurious falls than females before age 65, but lower rates on and after age 65.

**Figure 2:**
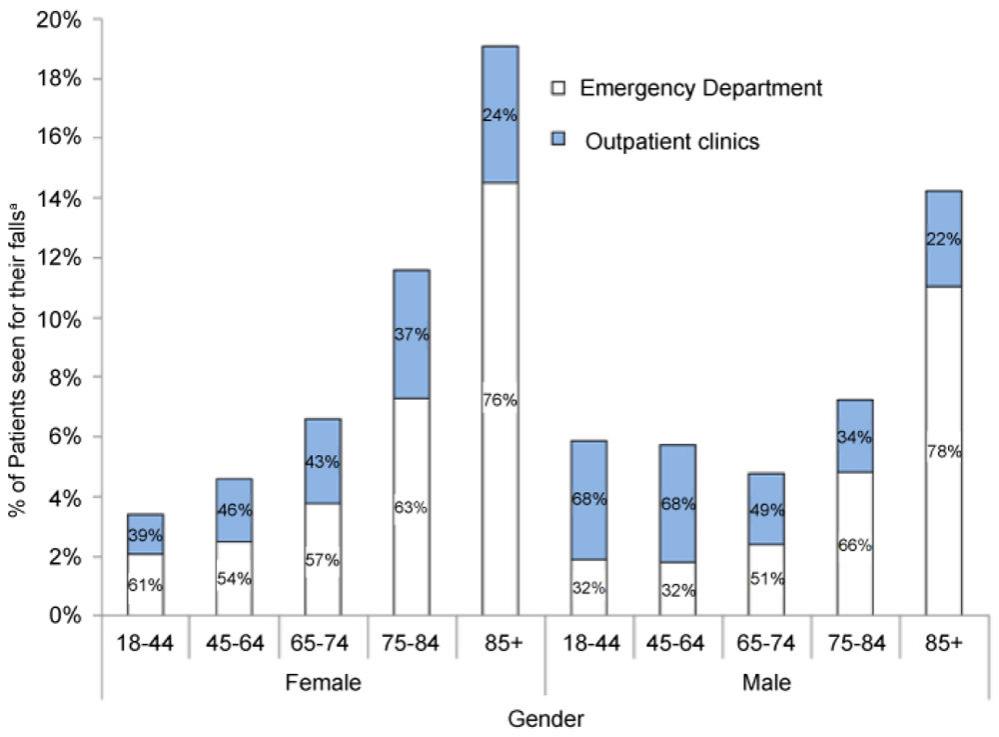
Percentage of patients of an academic medical center in the south central United States, who were seen for their falls^a^ in the emergency department versus outpatient clinics between 01/01/2007 and 06/30/2012, by gender and age.

To estimate the percentage of 14,016 medically consulted falls between January 2007 and June 2012 in this study that were treated in other points of entry outside the EDs, we used 2007-2011 NEISS-AIP data to estimate the rates of fall injuries treated in EDs. The NEISS-AIP rates of fall injuries treated in EDs for males of aged 18-44, 45-64, 65-74, 75-84, and 85+ years were 1.9%, 1.8%, 2.4%, 4.8%, 11.1%, respectively, and rates for females were 2.1%, 2.5%, 3.8%, 7.3%, 14.5%, respectively [1]. Figure 2 shows percentages of medical consulted falls treated at EDs versus other points of entry (outpatient clinics), by gender and age. We estimated that relying solely on ED data for medical consulted fall surveillance would fail to account for 22% (aged 85+ years) to 68% (aged 18-44 years) and 24% (aged 85+ years) to 46% (aged 45-64 years) of falls treated in men and women, respectively.

## Discussion

After taking into account falls treated in both EDs and outpatient clinics, medically consulted falls and injurious falls which resulted in head injuries or fractures increased with age for women. This however, was not true for men. In addition, women also had higher rates of medically consulted falls and injurious falls than men on and after age 65, but men had higher rates before age 65.

Previous studies found that older women were significantly more likely than older men to talk with a healthcare provider about falls and seek medical care [7]. Of unintentional fall injuries treated in EDs, fractures and head/neck injuries among older women were 2.2 and 1.3 times higher than for older men, respectively [4].

Malasana et al. [8] examined 6,043 patients presenting with a fall which resulted in total 8,163 visits to the University of Utah Health Care System between November, 2008, and October, 2009. Two-thirds of these visits were outpatient evaluations. The average payments received per fall patient evaluations were $3,200, resulting in an estimated yearly cost equal to $351,959,040.

In our study, over 76% of the oldest old (aged 85+ years) were treated in EDs, but the remaining didn’t go to EDs. Instead, they were treated at clinics for their falls. On the other hand, among those aged ≤ 84 years, only 32-78% of men and 54-76% of women were treated in EDs for their falls. Most strikingly, two-thirds of males aged 18-64 years didn’t go to EDs and were treated for their falls at an outpatient clinic instead.

The current study has several limitations. First, the number of medically consulted falls may be underestimated because it included only patients whose falls were treated at the study site Second, some falls may have been missed since we used ICD-9-CM diagnosis codes of E880-E888.xx to identify medically consulted falls. As with other studies utilizing E Codes it is difficult to know the completeness of the coding of records. Since E codes are supplementary codes that capture external causes of injury and are not mandatory [9], it is far more likely for these codes to be under-utilized rather than over utilized reducing the likelihood that our results are over estimated, but rather underestimated [10]. Despite these limitations, the primary strength of this study was using data from both ED and outpatient clinic visits to describe the gender differences in medically consulted falls and injurious falls that resulted in head injuries or fractures.

## Conclusion

Current findings demonstrate gender disparity of medically consulted falls and injurious falls resulting in head injuries or fractures among adult patients. Future research is needed to understand why gender disparity exists. Age and gender targeted fall injury prevention interventions need further development to prevent fall and reduce fall-related injuries. Significant fall-related injuries were treated at clinics only; future research is needed to determine whether fall injury surveillance should be expanded to include outpatient clinics.

## Figures and Tables

**Table 1:** Total number of patients seen for falls^a^ and injurious falls with head injuries or fractures^b^ in emergency department, inpatient and/or outpatient facilities at an academic medical center in the south central United States, between 01/01/2007 and 06/30/2012, by age.

Age	# Patients	Patients seen for their falls^a^	Patients seen for their injurious falls^b^
		No. (%)	No. (%)
18-44	121,653	5,240 (4.3)	1,501 (1.2)
45-64	86,820	4,413 (5.1)	1,517 (1.7)
65-74	28,152	1,632 (5.8)	548 (1.9)
75-84	16,664	1,623 (9.7)	583 (3.5)
85+	6,322	1,108 (17.5)	435 (6.9)
Total (%)	259,611	14,016 (5.4)	4,584 (1.8)

a Identified by International Classification of Diseases, 9th Revision, Clinical Modification (ICD-9-CM) diagnosis codes of E codes 880-888.

b Identified by ICD-9-CM diagnosis codes of E880-888 + primary diagnosis of intracranial injury: 850-854.xx, head Injuries: 959.01, fracture of skull: 800-804.xx, fracture of neck and trunk: 805-809.xx, fracture of upper limb: 810-819.xx, or fracture of lower limb: 820-829.xx.
